# Preliminary genomic characterisation and antimicrobial resistance of non-typhoidal *Salmonella* isolates from Burkina Faso within a One Health framework

**DOI:** 10.3389/fmicb.2026.1743556

**Published:** 2026-07-02

**Authors:** Namwin Siourimè Somda, Mah Alima Esther Traoré, Tomiwa Olumide Adesoji, Samuel Darkwah, Samiratu Mahazu, Patience B. Tetteh-Quarcoo, Eric S. Donkor

**Affiliations:** 1 Department of Medical Microbiology, University of Ghana Medical School, Accra, Ghana; 2 Département Technologie Alimentaire (DTA), Centre National de la Recherche Scientifique et Technologique (CNRST)/IRSAT, Bobo-Dioulasso, Burkina Faso; 3 Department of Microbiology, Obafemi Awolowo University, Ile-Ife, Nigeria

**Keywords:** antimicrobial resistance, Burkina Faso, genomic, non-typhoidal Salmonella, One Health approach

## Abstract

**Purpose:**

Non-typhoidal *Salmonella* (NTS) is a major foodborne pathogen worldwide, especially in low and middle-income countries. This study used whole-genome sequencing (WGS) to analyze the epidemiological trends, sequence types (STs), antimicrobial resistance (AMR), and genome dynamics, and to assess the phylogenetic relatedness of NTS isolates in Burkina Faso.

**Methods:**

A total of 34 presumptive *Salmonella enterica* isolates from animal products (*n* = 20), environmental sources (*n* = 8), and diarrheal stool samples (*n* = 6) were analyzed by Matrix-Assisted Laser Desorption Ionization-Time Of Flight mass spectrometry (MALDI–TOF MS). Isolates confirmed as *S. enterica* by MALDI–TOF MS were subsequently sequenced, and bioinformatic analysis was performed using the Bactopia pipeline.

**Results:**

Of the 34 presumptive *Salmonella* isolates, 21 (61.76%) were confirmed as *Salmonella enterica* by MALDI–TOF MS. Of the 21 *Salmonella enterica* detected, 16 were from animal products, 3 were from environmental sources, and 2 were from diarrheal stool samples. The most prevalent serovars were *S*. Schwarzengrund (ST96) and *S*. Give (ST516), each accounting for four isolates (19.04%). All isolates carried the resistance genes *emrR, pmrE, mdtK, cpxAR, bacA, baeSR, mdtABC, acrA, KdpE, golS, mdsABC, marA, mfd, msbA, sdiA, rob, UhpT* and *GlpT fosR*. One *S*. Molade or *S.* Wippra isolate ST544, harbored the AMR genes *fosA7-4, sul2, tet(A), dfrA14, qnrB1,* and *aph(3ʺ)-Ib.* In addition, one *S.* Llandoff isolate carried *catA, aac(6ʹ),* and *fosM*. Virulence genes, including *invA, avrA, iroB, iroC,* and *sinH,* were observed in all isolates, while 72.73% of the isolates harbored the *cdtB* gene.

**Conclusion:**

This is the first extensive study on non-typhoidal *Salmonella* and their clones in Burkina Faso. Effective AMR-inclusive surveillance strategies and novel control methods are needed to improve the management and treatment of multidrug-resistant (MDR) NTS infections and to mitigate the burden of NTS in the African sub-region.

## 1 Introduction

*Salmonella enterica* (*S. enterica*) is categorized into two serotype groups: typhoidal serotypes, which cause typhoid and paratyphoid fever, and nontyphoidal serotypes, which are responsible for human gastroenteritis (Plumb et al., 2023). These bacteria are generally found in environments with suboptimal hygienic conditions. Non-typhoidal *Salmonella* (NTS) is a major foodborne pathogen worldwide, especially in low- and middle-income countries (LMICs) (World Health Organization, 2018). It causes approximately 150 million illnesses and 60,000 deaths annually (Plumb et al., 2023). These deaths are frequently associated with therapeutic failure caused by increasing antimicrobial resistance in recent years (Tyson et al., 2021). Furthermore, NTS infections are generally linked to the consumption of contaminated animal-derived foods, water, and vegetables, as well as contact with infected individuals (Antunes et al., 2019).

NTS represents a major health burden in Africa, causing significant morbidity and mortality, particularly among young children and immunocompromised individuals, especially those living with HIV. The public health impact of NTS infections is particularly significant in Africa and Asia, where they have a profound effect on morbidity and mortality rates (Smith et al., 2016). In recent years, NTS has been reported to be responsible for a case fatality rate of more than 20% annually in sub-Saharan Africa among immunocompromised patients (Plumb et al., 2023). In Africa, data on the incidence and microbiology of non-typhoidal *Salmonella* remain limited and underreported. In addition, these estimates vary significantly from one region to another. In West Africa, the prevalence of NTS has been reported at 1.9 to 6.0% in Nigeria (Akinyemi et al., 2023; Aworh et al., 2024), 5.5 to 8.0% in Ghana (Archer et al., 2023; Dekker et al., 2019), and at 10.4, 17.9, and 18.9% in Burkina Faso (Kagambèga et al., 2021; Nikiema et al., 2021; Somda et al., 2021) from various sources. Moreover, self-medication among members of the population in LMICs to treat various infections is a common practice (Somda et al., 2021).

Furthermore, certain activities such as livestock farming, agriculture, and gardening increasingly rely on antibiotics for agropastoral production, amplifying the potential for the emergence and spread of resistant pathogens in LMICs (Jia et al., 2024; Kalule et al., 2025; Thindwa et al., 2019). The indiscriminate use of antibiotics, which are often available without a prescription, exacerbates the threat of AMR in these settings (Ahmad et al., 2024; WHO, 2024).

In Burkina Faso, as in most African countries, traditional microbiology techniques are primarily used for pathogen identification, although there has been progress in building genomic capacity in the aftermath of the COVID-19 pandemic (Héma et al., 2023; Somda et al., 2025). However, some of this infrastructure has remained non-functional due to a lack of qualified technical personnel (Somda et al., 2025). Genomic data on multidrug-resistant *Salmonella* in sub-Saharan Africa are scarce and poorly documented, creating challenges in establishing robust surveillance frameworks for these pathogens (Somda et al., 2025). Recently, modern techniques such as whole-genome sequencing (WGS) and multilocus sequence typing (MLST) have become common approaches for microbial typing to determine bacterial clones, antibiotic resistance genes, evolutionary relationships, and distributions across hosts and environments (Achtman et al., 2012). In Burkina Faso, data regarding the genomic diversity and multidrug resistance of NTS are often insufficient. This report is the first in Burkina Faso to assess the genomic diversity of NTS from a One Health approach. To address this gap, this study used WGS to analyze the epidemiological trends, sequence types (STs), antimicrobial resistance (AMR), and genome dynamics, to assess the phylogenetic relatedness of NTS in Burkina Faso. The results will contribute to elucidating the genomic diversity of *Salmonella enterica* populations in human, environmental, and animal hosts, unraveling the population structure, burden, and evolution of AMR in these hosts within a One Health context. Furthermore, these findings will contribute to understanding the mechanisms underlying AMR in pathogens of public health interest, particularly in Burkina Faso, where they will improve the surveillance of AMR.

## 2 Methodology

### 2.1 Study design

*Salmonella* *enterica* was recovered in Burkina Faso from 826 samples, including 498 animal products (200 liver, 200 cecal content, and 98 mesenteric lymph node samples), 135 samples from environmental sources [comprising lettuce (*n* = 45), irrigation water (*n* = 45), and organic manure (*n* = 45)], and 193 diarrhea stool samples. All non-duplicate *S. enterica* clinical isolates were collected between September 2019 and March 2020, animal product isolates between October 2021 and January 2022, and environmental isolates between March and December 2023.

### 2.2 Microbiology analyses

A total of 34 presumptive isolates of *Salmonella enterica—*including 20 from animal products, 8 from environmental sources, and 6 from clinical samples—were identified using standard microbiological methods and stored at −20°C for further analysis. All suspected *Salmonella* isolates were cultured on Mueller-Hinton agar (Liofilchem S.r.l., Italy) and incubated at 37°C for 18–24 h. Thereafter, specific colonies were subjected to biochemical profiling using the API 20E system according to the manufacturer’s instructions (BioMérieux, France) for further confirmation. Confirmed isolates were stocked in sterile cryotubes containing brain heart broth (BHB) supplemented with 15% glycerol and stored at −20°C for MALDI–TOF MS and molecular analysis.

### 2.3 Matrix-assisted laser desorption ionization-time of flight mass spectrometry (MALDI–TOF MS)

The presumptive *Salmonella* isolates were cultured on Mueller-Hinton agar (Liofilchem S.r.l., Italy) and incubated at 37 °C for 18–24 h. Isolates were identified using MALDI–TOF MS according to the manufacturer’s instructions (BioMérieux, France) (Bizzini et al., 2010). MALDI–TOF MS identification was performed on colonies of interest isolated from culture media. Individual colonies from overnight cultures were suspended in 300 μL of double-distilled water and mixed with 900 μL of 96% ethanol (Carl Roth GmbH, Karlsruhe, Germany) for precipitation. After centrifugation for 5 min at 10,000 × *g*, the supernatant was removed, and the pellet was resuspended in 50 μL of 70% (vol/vol) formic acid (Sigma-Aldrich Chemie GmbH, Steinheim, Germany). Subsequently, 50 μL of acetonitrile (Carl Roth GmbH) was added, mixed, and centrifuged for 5 min at 10,000 × *g*. A 1.5 μL aliquot of the supernatant was transferred onto a microtiter plate format (MTP) 384 target plate polished steel transponder/tracking (TF) (Bruker Daltonik GmbH, Bremen, Germany). After air-drying, the material was overlaid with 2 μL of a saturated solution of *α*-cyano-4-hydroxycinnamic acid (Sigma-Aldrich Chemie GmbH) in a mix of 50% acetonitrile and 2.5% trifluoroacetic acid (Sigma-Aldrich Chemie GmbH). Once dried, mass spectra were acquired using an Ultraflex II instrument (Bruker Daltonik GmbH). The instrument was calibrated using the Bacterial Test Standard (Bruker Daltonik GmbH). Data analysis was performed using Biotyper 4.1.100 (PYTH) software (Bruker Daltonik GmbH). The results were interpreted according to the manufacturer’s recommendation: a score of ≥ 2.3 represented reliable species-level identification; a score of 2.0–2.29 represented probable species-level identification; a score of 1.7–1.9 represented probable genus level identification; and a score ≤ 1.7 was considered an unreliable identification (Lüthje et al., 2017). Finally, pure cultures of the confirmed isolates were used for deoxyribonucleic acid (DNA) extraction.

### 2.4 Whole-genome sequencing

The whole-genome sequencing (WGS) of the isolates was performed at the West African Centre for Cell Biology of Infectious Pathogens (WACCBIP) located within the University of Ghana campus. In brief, genomic DNA for short-read sequencing was isolated from overnight cultures of all *Salmonella enterica* isolates (*n* = 21) using the QIAamp DNA Mini kit (Qiagen, Hilden, Germany) according to the manufacturer’s instructions. DNA concentrations were quantified using the Qubit 4.0 Fluorometer Assay Kit (Thermo Fisher Scientific, Waltham, MA, United States). DNA libraries were prepared using the Illumina DNA prep kit (Illumina Inc., San Diego, CA, United States) and sequenced on an Illumina NextSeq 1000/2000 platform using the NextSeq 1000/2000 P2 Reagent Kit (300 cycles).

### 2.5 Bioinformatics analysis: data analysis

Comprehensive processing of the sequence reads—including quality control steps, assembly, annotation—was executed using the Bactopia pipeline (Petit 3rd and Read, 2020). This Nextflow-based pipeline enables comprehensive and standardized processing of bacterial genomic data.

Quality control of reads: Initial quality control of the raw reads was conducted using FastQC,^1^ which was used to analyze quality scores, base content, and duplications (Simon Andrews et al., 2010). Fastp^2^ was used for filtering and was subsequently employed to filter low-quality reads, remove adapters, and perform automatic correction (Chen et al., 2018). These steps ensured the quality and integrity of the data prior to assembly. The cleaned reads were assembled using Shovill, a Bactopia-integrated tool that utilizes the SPAdes assembler optimized for the rapid assembly of Illumina reads^3^ (Bankevich et al., 2012). Assembly quality was evaluated using the Quality Assessment Tool for Genome Assemblies (QUAST),^4^ to determine metrics such as the number of contigs, N50, total size, and GC content (Gurevich et al., 2013). Functional genome annotation was performed using the following tool: Prokka,^5^ a fast and accurate tool for annotating bacterial genomes (Seemann, 2014). The MLST^6^ scheme specific to *Salmonella* was used to determine the sequence types (STs) of the isolates. AMRFinderPlus integrated within Bactopia,^7^ was utilized to identify antibiotic resistance genes (ARGs), virulence genes, and resistance-associated point mutations (Feldgarden et al., 2019). In silico typing resource (SISTR v1.1.2)^8^ and SeqSero2 v1.3.1^9^ were used for *Salmonella* strains serotyping (Watts and Holt, 2019). A core-genome gene alignment was generated using Roary v3.13.0 and the Multiple Alignment using Fast Fourier Transform (MAFFT) aligner (Page et al., 2015). Maximum-likelihood phylogenetic trees were constructed using IQ-TREE v3.0.1,^10^ applying the General Time Reversible + Gamma (GTR + G) nucleotide substitution model (Nguyen et al., 2015). Node support was rigorously assessed using 1,000 ultrafast bootstrap replicates and 1,000 Shimodaira–Hasegawa-like approximate likelihood ratio (SH-aLRT) tests.

To define phylogenetic clusters objectively, a pairwise single-nucleotide polymorphism (SNP) distance matrix was computed from the core-gene alignment using snp-dists v0.8.2. To minimize visual bias, this matrix was subjected to hierarchical clustering using the unweighted pair group method with arithmetic mean (UPGMA) algorithm (average linkage) implemented in SciPy v1.17.0. The optimal number of clusters was determined analytically by calculating silhouette scores for different values of *k* using scikit-learn v1.8.0. Heatmaps visualizing the presence/absence of resistance and virulence genes (Kolde, 2019) were generated using the Pheatmap in R package tool. Finally, the ggtree R package was used for visualizing and annotating phylogenetic trees (Yu et al., 2017).

## 3 Results

### 3.1 Bacteria isolates

Of the 826 samples analyzed in this study, 34 presumptive *Salmonella* (4.11%)—including 20 from animal products, 8 from environmental sources, and 6 from clinical samples—were identified using the API 20E system (Table 1). Among the animal product samples, 14 presumptive *Salmonella* isolates were detected in cecal contents, 3 isolates each in mesenteric lymph nodes and liver samples (Table 1). Of the 34 presumptive *Salmonella* isolates, 21 (61.76%) were identified as *Salmonella enterica* using MALDI–TOF MS (Table 2). Out of 21 *Salmonella enterica* identified, 3.21% (16/498) were detected in animal products, 2.22% (3/135) in environmental samples, and 1.04% (2/193) from clinical samples. Geographically, all isolates recovered from animal products (*n* = 16) were sourced from Nanoro, while the clinical (*n* = 2) and environmental (*n* = 3) isolates originated from Ouagadougou and Bobo-Dioulasso, respectively. Among the animal sources, *Salmonella enterica* was most frequently recovered from goats (*n* = 6) (Table 2).

**Table 1:** Distribution of *Salmonella enterica* isolates in animal products after biochemical identification and MALDI-TOF confirmation.

Products	Animal	Donkey	Goat	Sheep	Guinea fowl	Pork	Chicken	Total
Caecal contents (N/Ech)		3/30	3/85	2/11	2/27	2/11	2/36	14/200
Liver (N/Ech)		0/30	3/85	0/11	0/27	0/11	0/36	3/200
Mesenteric ganglion (N/Ech)		0	2/85	0/3	0/3	1/4	0/3	3/98
Total (N/Ech)		3/60	8/255	2/25	2/57	3/26	2/75	20/498

N/Ech, number of isolates after biochemical identification/number of samples.

**Table 2:** Distribution of *Salmonella enterica* isolates in animal products after MALDI-TOF confirmation.

Products	Animal	Donkey	Goat	Sheep	Guinea fowl	Pork	Chicken	Total
Caecal contents (N/Ech)		2/30	3/85	1/11	2/27	2/11	2/36	12/200
Liver (N/Ech)		0/30	2/85	0/11	0/27	0/11	0/36	2/200
Mesenteric ganglion (N/Ech)		0	1/85	0/3	0/3	1/14	0/3	2/98
Total (N/Ech)		2/60	6/255	01/25	02/57	3/26	2/75	16/498

N/Ech, number of isolates after MALDI-TOF confirmation/number of samples.

### 3.2 Whole-genome sequencing (WGS) and bioinformatic analysis

Whole‑genome sequencing revealed that 21 isolates were confirmed as *Salmonella enterica* and were assigned to 12 different serovars. The identified serovars included *S*. Schwarzengrund (*n* = 4), *S*. Give (*n* = 4), *S*. Llandoff (*n* = 2), and *S*. Sundsvall (*n* = 2), alongside singletons of *S*. Gokul, *S*. Irenae, *S*. Molade or Wippra, *S*. Poona, and *S*. Tennessee. However, the serovars of three isolates remained undetermined (Table 3). In addition, the isolates were assigned to seven sequence types, including ST96 (*n* = 4), ST516 (*n* = 4), and ST488 (*n* = 2). Others included singletons of ST544, ST2609, ST5440, and ST6879. However, seven of the isolates were not assigned to any known sequence type (Table 2; Supplementary Table S1). Notably, the *S.* Schwarzengrund serovar was recovered from animal, human, and environmental samples, whereas *S.* Give was obtained only from animal and environmental samples.

**Table 3:** Distribution of Non-Typhoidal *Salmonella* serotypes isolated from One Health approach.

Isolate ID	Location	Source	Isolates	Antigenic profile	Serovars	MLST
0146/X/CHECC	Nanoro	Goat	*Salmonella enterica*	6,14:z:e,n,x	Sundsvall	ST488
059/KUN/F2	Bobo	Organic fertilizer	*Salmonella enterica*	3,10:l,v:1,7	Give	ST516
041ANE CC	Nanoro	Donkey	*Salmonella enterica*	39:k:1,6	I 39:k:1,6	ND
124PINCC	Nanoro	Guinea fowl	*Salmonella enterica*	7:m,t:-	Oranienburg	ND
078PORCC E	Nanoro	Pork	*Salmonella enterica*	3,10:l,v:1,7	Give	ST516
078PORCC F	Nanoro	Pork	*Salmonella enterica*	3,10:l,v:1,7	Give	ST516
COPRO I 20	Ouaga	Stools	*Salmonella enterica*	47:z4,z23:-	I 47:z4,z23:-	ND
Y SIN C1	Ouaga	Stools	*Salmonella enterica*	4:d:1,7	Schwarzengrund	ST96
156 PORC C	Nanoro	Pork	*Salmonella enterica*	51:d:1,5	Gokul	ST5440
CHEG 179	Nanoro	Goat	*Salmonella enterica*	-:-:-	- -:-:-	ND
042CHECC	Nanoro	Goat	*Salmonella enterica*	6,14:z:e,n,x	Sundsvall	ST488
061POUCC	Nanoro	Chicken	*Salmonella enterica*	1,3,19:z29:-	Llandoff	ND
007CHEG E	Nanoro	Goat	*Salmonella enterica*	3,10:l,v:1,7	Give	ST516
067POUCC	Nanoro	Chicken	*Salmonella enterica*	1,3,19:z29:-	Llandoff	ND
004CHEG F	Nanoro	Goat	*Salmonella enterica*	4:d:1,7	Schwarzengrund	ST96
12PINCC	Nanoro	Guinea fowl	*Salmonella enterica*	4:d:1,7	Schwarzengrund	ST96
142ANE CC	Nanoro	Donkey	*Salmonella enterica*	13:z:1,6	Poona	ST2609
MLE V1 RVS SS	Bobo	Lettuce	*Salmonella enterica*	8:z10:z6	Molade or Wippra	ST544
80MOUCC	Nanoro	Sheep	*Salmonella enterica*	7:z29:-	Tennessee	ND
168CHEG H	Nanoro	Goat	*Salmonella enterica*	17:k:1,5	Irenea	ST6879
021/Kdn/F03	Bobo	Organic fertilizer	*Salmonella enterica*	4:d:1,7	Schwarzengrund	ST96

ND, Undetermined.

The prevalence of AMR genes was investigated in the *Salmonella enterica* isolates. All isolates contained the multidrug efflux transporter *mdsA/mdsB*. One *S*. Molade or Wippra ST544 isolate recovered from lettuce in Bobo-Dioulasso harbored the antibiotic resistance genes *fosA7-4, sul2, tet(A), dfrA14, qnrB1,* and *aph(3″)-Ib,* which confer resistance to fosfomycin, sulfonamide antibiotics, such as sulfamethoxazole, tetracycline, trimethoprim, fluoroquinolone, and aminoglycosides, respectively. This isolate also harbored *aph(6′)-Id,* an aminoglycoside phosphotransferase encoded by plasmids, and integrative conjugative elements. One of the *S.* Llandoff serovar isolated from chicken in Nanoro harbored the antibiotic resistance genes, *catA, aac(6′),* and *fosM*, which confer resistance to chloramphenicol, aminoglycoside, and fosfomycin, respectively.

All isolates analyzed in this study carried the resistance genes *emrR, pmrE, mdtK, cpxAR, bacA, baeSR, mdtABC, acrA, KdpE, golS, mdsABC, marA, mfd, msbA, sdiA, UhpT fosR, rob*, and *GlpT fosR*, which are associated with resistance to multiple classes of antibiotics. Among the isolates, strain SN17 contained all the antimicrobial resistance genes identified through genome analysis using the Comprehensive Antibiotic Resistance Database (CARD). The *aac(6′)-Iy* gene which encodes an aminoglycoside acetyltransferase enzyme that provides bacteria with resistance to specific aminoglycoside antibiotics (including tobramycin, amikacin, and netilmicin), was detected in 20/21 isolates. Conversely, the *fosA7* and *vgaC* genes, which confer resistance to fosfomycin and to streptogramin A and lincosamides, respectively, were each detected in only two isolates (Supplementary Table S2). The *qnrB5* and *aac(6′)-Iaa* genes were also each detected in a single isolate.

The presence of virulence genes was also investigated in the *Salmonella enterica* isolated in Burkina Faso. It was observed that all the *Salmonella* isolates carried at least one virulence gene. Specifically, the virulence genes; *invA, avrA, iroB, iroC* and *sinH* were identified in all isolates (100%), followed by *cdtB* in 72.73% of the isolates, and *lpfB* and *ssek2* at a lower frequency of 22.73% each. It was observed that four of the isolates carried seven different virulence genes. Overall, this study highlighted three specific strains, namely a clinical *S*. Schwarzengrund isolate, an *S*. Llandoff isolate from chicken, and an *S*. Molade or Wippra isolate from lettuce, that harbored several resistance and virulence genes.

**Figure 1:**
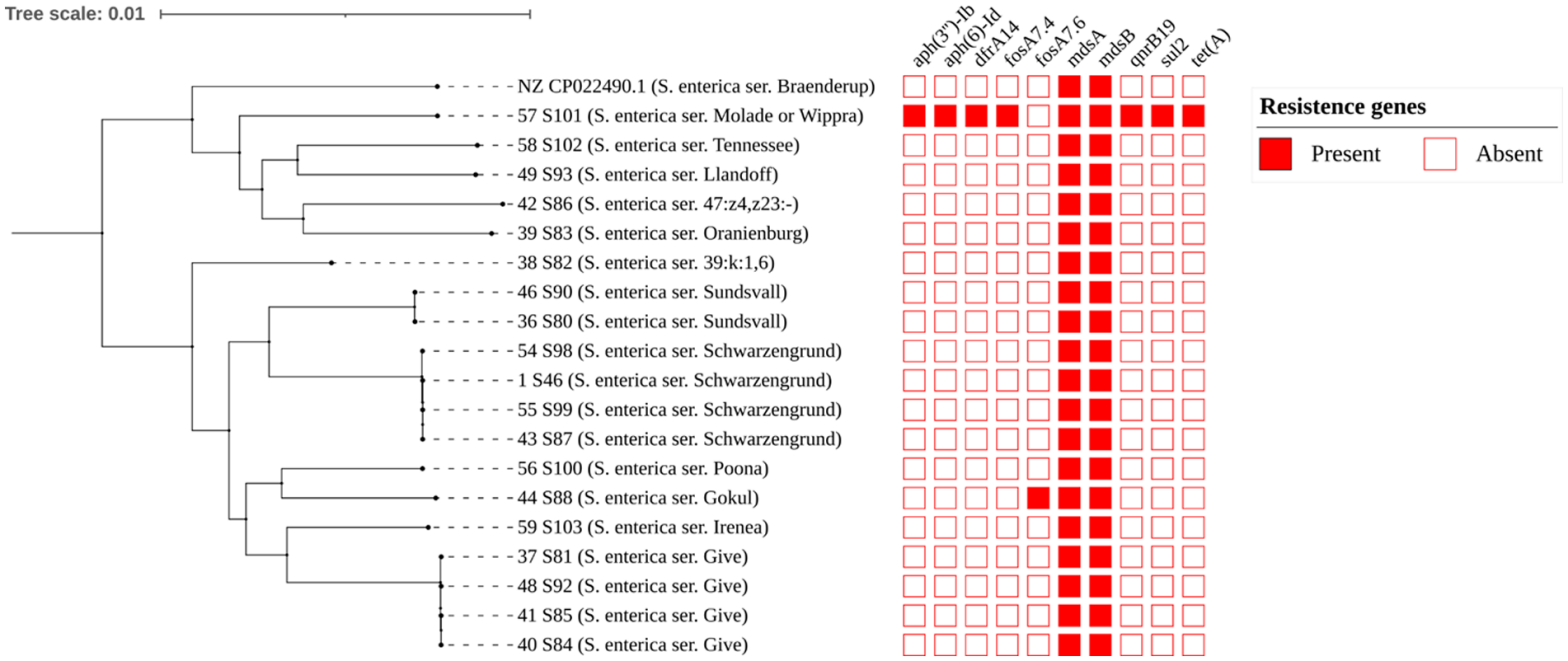
Distribution of non-typhoidal *Salmonella* serovars and resistance genes.

**Figure 2:**
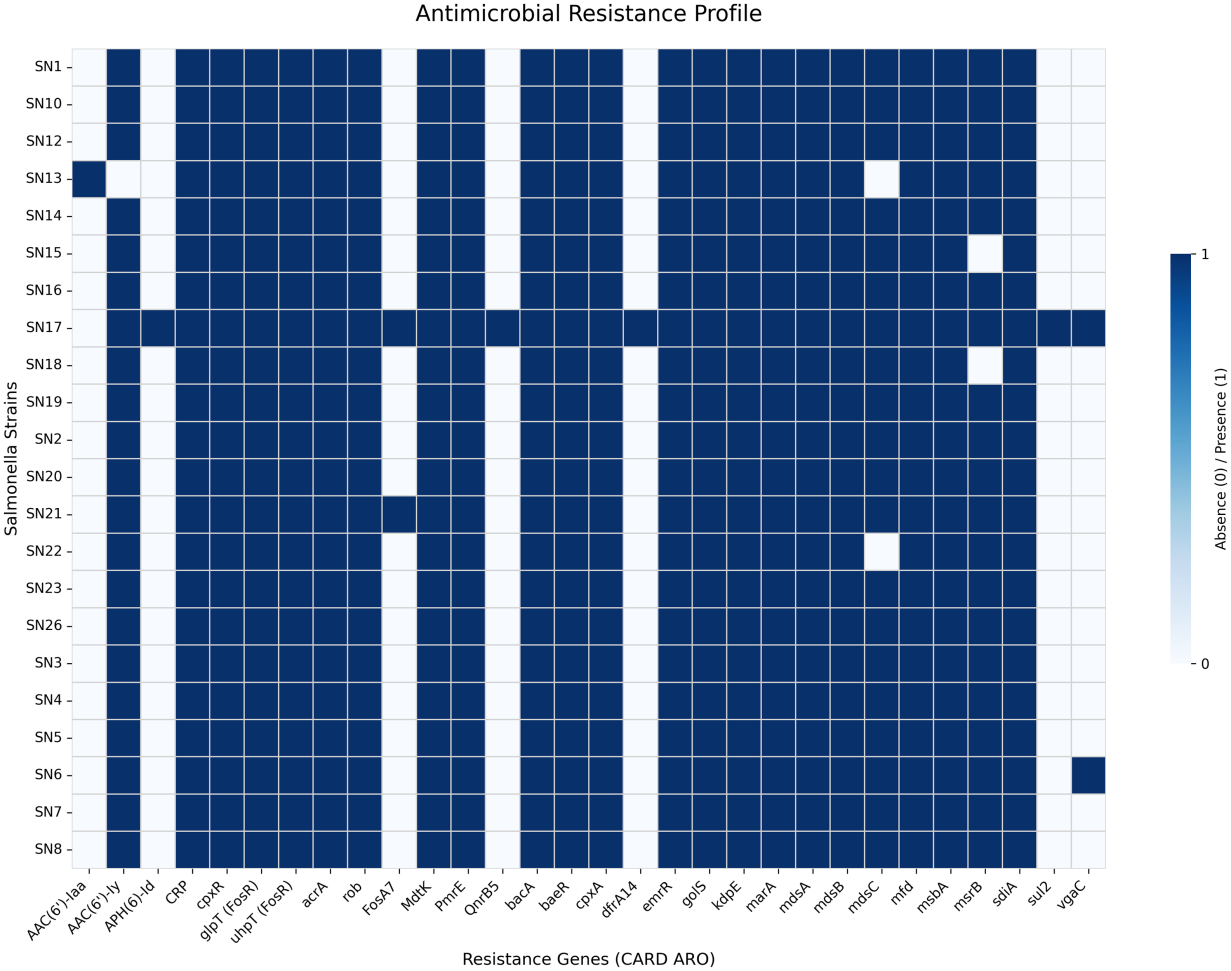
Heatmap of antimicrobial resistance genes identified by whole-genome analysis using the Comprehensive Antibiotic Resistance Database (CARD).

**Figure 3:**
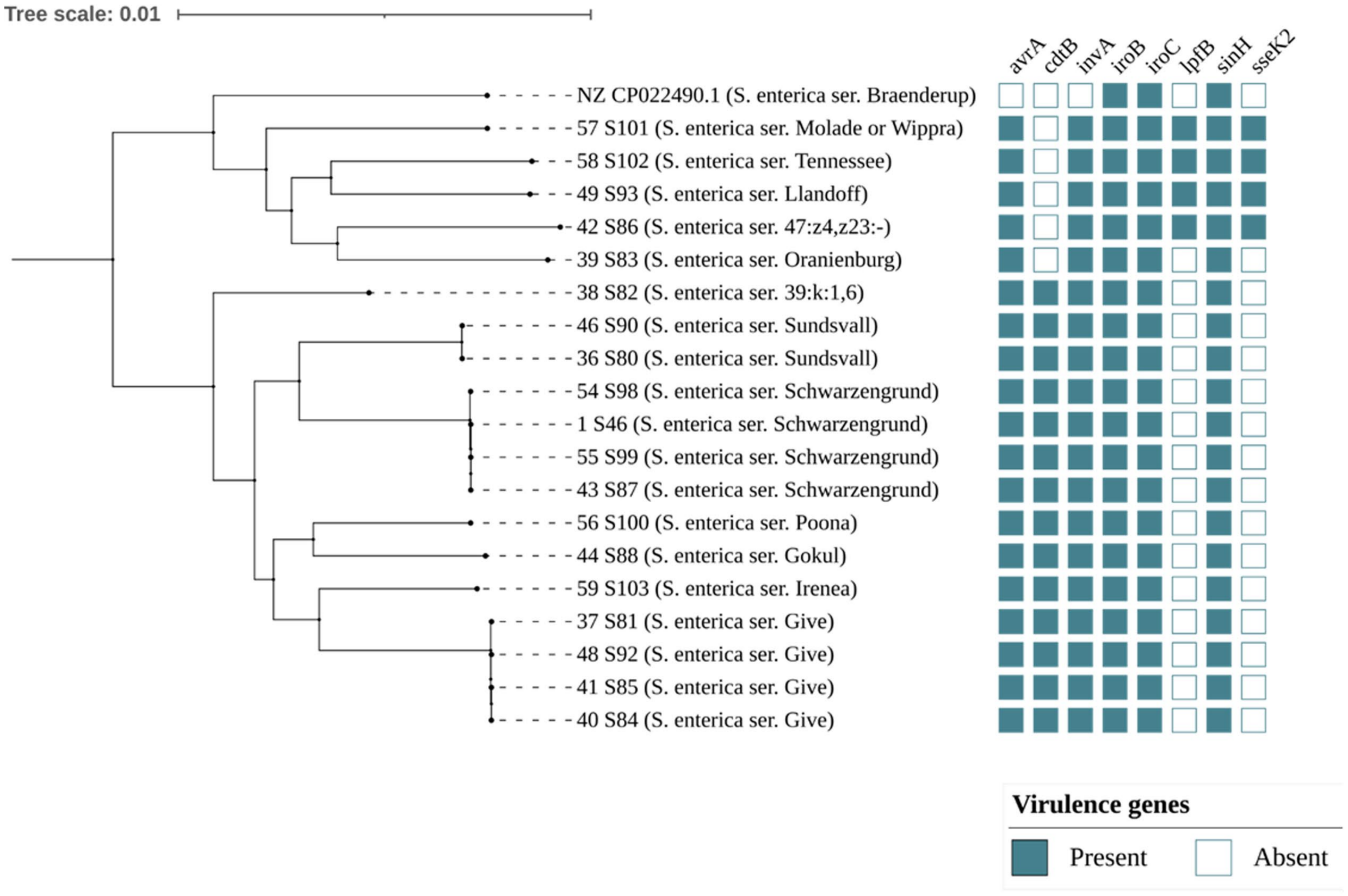
Virulence genes detected in non-typhoidal *Salmonella* isolates recovered from human, environmental, and animal product sources.

**Figure 4:**
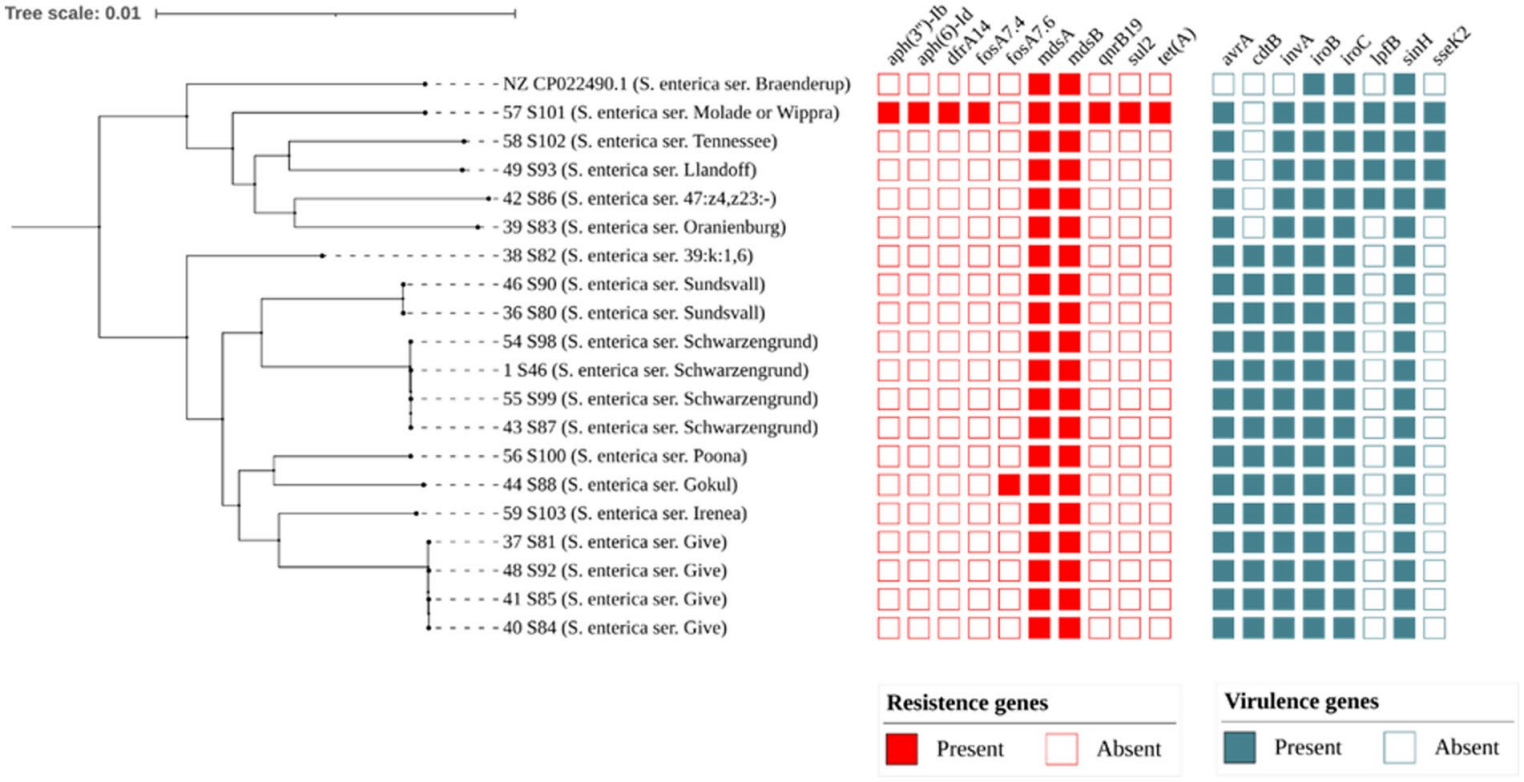
Distribution of multidrug-resistant and virulent *Salmonella* isolates from a one health perspective in Burkina Faso.

In this study, MLST revealed four distinct clusters comprising 13 *S. enterica* strains (Supplementary Figure S1). Analysis of the phylogenetic tree showed that nine isolates did not cluster, including *S*. Irenae, *S*. Poona, *S*. Gokul, *S*. I 39:k:1,6, *S*. Tennessee, *S*. Molade or Wippra, *S*. I 47:z4,z23:-, and *S*. Oranienburg. However, one *Salmonella* isolate was non-typeable and was therefore not represented on the tree. Cluster 1 was represented by *S*. Schwarzengrund (ST96) and comprised four *Salmonella* isolates, one from diarrheal stool in Ouagadougou, two from animal products (goat and guinea fowl) in Nanoro and one from organic fertilizer in Bobo-Dioulasso. Cluster 2 consisted of *S*. Give (ST516) and included four *Salmonella* isolates, including two from pork in in the rural area of Nanoro and one from organic fertilizer used in gardening in Bobo-Dioulasso. Cluster 3 (*S*. Sundsvall, ST488) included two *Salmonella* isolates originating from goat in Nanoro, while Cluster 4 (*S*. Llandoff, STND) consisted of two strains from chickens in Nanoro (see Figures 1–4).

## 4 Discussion

*Salmonella* can be isolated from various sources, including food, water, soil, animals, and production equipment, with the frequency of detection depending on the type of product. In this study, *Salmonella enterica* was isolated from 3.21, 2.22, and 1.04% of animal products, environmental sources, and clinical samples. Previous investigations conducted in Burkina Faso have varying prevalence rates for *Salmonella enterica* across various sample types. For instance, Nikiema et al. (2021) reported a 17.9% prevalence in contaminated street foods, Kagambèga et al. (2021) found an 18.9% prevalence in imported frozen fish, and Somda et al. (2021) reported a 31.34% prevalence in lettuce and irrigation water alongside a 3.6% prevalence in human clinical samples. The elevated presence of these bacteria in prior reports has been attributed to poor hygienic and storage conditions leading to food contamination. This factor could explain the high prevalence of *Salmonella* in previous studies, in contrast to the results obtained in this study. Furthermore, the highest prevalence of *Salmonella* reported by Somda et al. compared to our results in environmental samples is unsurprising given that their samples comprised wastewater from hospital drainage channels used for vegetable irrigation, while our samples were rainwater used for garden irrigation.

Among the *Salmonella* isolates in this study, it was observed that the serovar *S*. Schwarzengrund, ST96, was one of the most prevalent. This is the second study reporting the ST96 sequence type in Burkina Faso after a study conducted in 2019 by Post et al. (2019). This serotype has been reported in several other African countries including Malawi, Nigeria, Senegal and Tunisia (Dieye et al., 2022; Jibril et al., 2020; Rigby et al., 2025; Soufi et al., 2012). Globally, ST96 has also been reported in Indonesia from chicken meat (Takaichi et al., 2022) and in the food production chain in Brazil (Monte et al., 2019). According to Ikeuchi et al. (2024), *S*. Schwarzengrund ST96 has been recognized in recent years as a foodborne pathogen transmitted from animals to humans—particularly via contaminated chicken in Japan—leading to non-typhoidal salmonellosis (Ikeuchi et al., 2024). Studies indicate that it is a prevalent non-typhoidal serovar with increasing detection in poultry and the potential for widespread geographic expansion (Noenchat et al., 2023; Yamazaki et al., 2016). Several factors could be linked to the frequent presence of this serotype in our sub-region, notably the importation of broiler chickens into local markets. It is worthy of note that the ser. *S*. Schwarzengrund, ST96, isolates in this study were recovered from animal, humans and environmental samples.

Our findings reveal that *S.* Give, ST516, was also one of the most prevalent serotypes detected. The isolates were obtained from animals (pork and goat) and environmental samples (organic fertilizer). This observation is not surprising, as the insufficiency of mineral matter in the soil and the inability of certain farmers to afford chemical fertilizers have led to the widespread use of organic manure for garden soil fertilization in Burkina Faso. This manure usually consists largely of animal feces, particularly from pigs, poultry, and goats (Somda et al., 2021). *S.* Llandoff and *S.* Sundsvall were isolated from chicken and goat, respectively. *Salmonella* serotypes *S*. Llandoff and *S*. Sundsvall have been reported to be transmitted through the consumption of contaminated food and water, with primary reservoirs in animals such as poultry, pigs, fish, and cattle. The bacteria can spread from animals to humans through the farm-to-fork continuum, most commonly via animal-derived products (Antunes et al., 2017; Henry et al., 2012). Data on the circulation of the other serotypes reported in this study (*S*. Gokul, *S*. Irenae, *S*. Molade or Wippra, *S*. Poona, and *S*. Tennessee) are scarce and not documented in Burkina Faso. The fact that the data about these different serotypes are limited and not documented in Burkina Faso does not necessarily mean that these serotypes are not circulating in Burkina Faso. Previous studies have reported the presence of *Salmonella* in Burkina Faso. However, these studies did not include serotyping of the *Salmonella* isolates (Compaoré et al., 2020; Rouamba et al., 2022; Soubeiga et al., 2022; Tiendrebeogo et al., 2024). Many of these studies have not used serotyping or whole-genome sequencing, as these modern tools are expensive. Low research budget in developing countries could be a limiting factor. It will be necessary to extend further studies to other animals using these modern genomic tools to determine the possible host range of non-typhoidal *Salmonella* strains in Africa. These findings support the necessity of the “One Health” concept in all African countries, particularly when investigating zoonotic pathogens such as *Salmonella*. In addition, certain isolates were not assigned to any sequence type. The occurrence of unassigned (ND) serotypes and sequence types (STs) could be explained by the presence of previously unreported variants or alleles absent from existing MLST or SeqSero2 databases, or by inadequate genome assembly quality for accurate typing.

The detection of a broad repertoire of resistance-associated genes in all isolates suggests that these bacteria possess a highly conserved and multifaceted AMR background. The identified genes are involved in several complementary resistance mechanisms, including multidrug efflux, membrane modification, stress-response regulation, and reduced antibiotic uptake, indicating a strong adaptive capacity against diverse antimicrobial agents.

A major finding is the universal presence of multiple multidrug efflux systems and their regulators, including *mdtABC, mdsABC, acrA, mdtK, marA, rob, baeSR*, and *emrR*. These genes are known to contribute to the active extrusion of structurally unrelated antibiotics from bacterial cells (Qing et al., 2024). According to Alav et al. (2022), the *AcrAB*-associated system, represented here by *acrA*, is one of the most important efflux pumps in Gram-negative bacteria and is linked to resistance against *β*-lactams, fluoroquinolones, tetracyclines, chloramphenicol, and other compounds. Similarly, *mdtABC*, *mdsABC*, and *mdtK* encode transporters that enhance tolerance to toxic substances and antimicrobials (Qing et al., 2024). Regulatory genes such as *marA, rob*, and *baeSR* can globally activate efflux pump expression and stress-response pathways, thereby amplifying multidrug resistance phenotypes.

The identification of *cpxAR, KdpE, golS*, and *sdiA* further indicates that the isolates possess sophisticated regulatory systems that enable adaptation to hostile environmental conditions, including antibiotic exposure. According to Jing et al. (2021), the *CpxAR* two-component system is particularly important because it responds to envelope stress and has been associated with reduced susceptibility to several antimicrobial classes. Likewise, *golS* contributes to heavy metal tolerance and can indirectly co-select for antibiotic resistance, while *sdiA* participates in quorum sensing and the regulation of virulence and stress responses (Martins et al., 2022). These findings suggest that resistance in these isolates is not limited to isolated resistance determinants but instead involves coordinated regulatory networks that enhance bacterial survival.

Genes associated with cell envelope modification and intrinsic resistance were also consistently detected. Joo et al. (2023) showed that the presence of *pmrE* and *bacA* is noteworthy because these genes are involved in lipopolysaccharide and membrane-associated modifications that reduce susceptibility to antimicrobial peptides and certain antibiotics. *MsbA*, an ATP-binding cassette transporter involved in lipid A transport and membrane biogenesis, may further contribute to decreased intracellular antibiotic accumulation. Such membrane-associated resistance mechanisms are important because they can provide baseline protection against multiple antimicrobial agents.

In this study, the presence of *mfd*, a gene involved in DNA repair and mutation processes, is also significant. Beyond its role in transcription-coupled DNA repair, *mfd* has been linked to accelerated mutation rates and the evolution of antimicrobial resistance under selective pressure (Guillemet et al., 2016). Its conservation among all isolates may enhance bacterial adaptability and facilitate the emergence of additional resistance traits over time.

Overall, the uniform distribution of these genes across all isolates suggests the existence of a conserved resistome that may reflect strong and prolonged selective pressure from antimicrobial exposure in the environment, clinical settings, or agricultural systems. Importantly, many of these determinants are associated with intrinsic resistance or global regulatory pathways rather than acquired resistance alone, implying that the isolates may possess both baseline and inducible resistance capabilities. The coexistence of multiple resistance mechanisms within the same isolates raises concern because synergistic interactions among efflux systems, membrane modifications, and regulatory networks can substantially reduce antibiotic efficacy and complicate treatment strategies.

All *Salmonella* isolates harbored the multidrug efflux transporter *mdsA/mdsB* genes. Only one *S*. Llandoff isolate from chicken and one *S*. Molade or Wippra isolate from an environmental sample exhibited multidrug resistance (MDR). These isolates harbored genes conferring resistance to chloramphenicol, fosfomycin, macrolides, sulfamethoxazole, tetracycline, trimethoprim, fluoroquinolone, and aminoglycosides and were identified at two distinct locations. According to Wang et al. (2025), the geographic pattern of AMR varied depending on the host/source considered. They also indicated that the geographic pattern of AMR varied depending on the classes of antimicrobials considered. Many studies conducted in African countries have shown similar results (Akinyemi et al., 2023; García-Díez et al., 2024). These antibiotics are widely and always available, inexpensive, and commonly used in humans without prescription in Burkina Faso. In this study, the fosfomycin resistance gene was detected in one clinical isolate and one isolate from a goat. The dissemination of this gene is not unexpected, as fosfomycin has recently been reintroduced into clinical practice, particularly for the treatment of infections caused by extended-spectrum beta-lactamase (ESBL)-producing Enterobacteriaceae, due to the limited availability of new effective antimicrobials (Falagas et al., 2016). These findings are consistent with those reported by Wang et al. (2023a, 2023b).

According to Somda et al. (2024), animals and the environment are considered the fastest route for the transmission and dissemination of *Salmonella* and AMR genes. Antibiotics are employed in intensive livestock farms, as therapeutics and animal growth promotion agents, which selects for resistant bacteria and result in the presence of antibiotic residues in farming effluents (Kedišaletše et al., 2023). The variation in AMR patterns by geography, host/source, and antimicrobial class reflects a complex interplay of antibiotic use, environmental conditions, and bacterial genetics. This underscores the importance of a One Health approach, integrating human, animal, and environmental data to better understand and control AMR (Wang et al., 2025). These findings underscore the importance of continuous genomic surveillance and antimicrobial stewardship, as isolates harboring such extensive resistance-associated determinants may serve as reservoirs for the dissemination of multidrug resistance within bacterial populations.

None of the isolates possessed the ESBL or carbapenem resistance genes. This could be explained by the fact that these antibiotics are not accessible because they are expensive and not readily available to rural dwellers. This finding is similar to the report by Akinyemi et al. (2023), who reported the presence of *Salmonella* from stool, cattle, poultry, feces, and wastewater samples in Nigeria. In addition, reports by Hugho et al. (2024), who detected *Salmonella* from diarrhea, domestic animal feces, food, and water samples in Tanzania, and Mattock et al. (2022), who detected the pathogen from various samples in South Africa, are similar to the present report. In contrast, studies from China have documented the presence of ESBL and carbapenem resistance genes in *Salmonella* isolates (Wang et al., 2023a; Wang et al., 2025; Wang et al., 2023b). This discrepancy may reflect lower antibiotic pressure, a different epidemiological setting, and the limited dissemination of resistant clones in Burkina Faso. However, ongoing globalization and increased antibiotic use may facilitate their future emergence, emphasizing the importance of continuous surveillance using a One Health approach.

All *Salmonella enterica* isolates possess an arsenal of virulence factors that play important roles at different stages of infection. In this study, all NTS isolated harbored *invA, avrA, iroB, iroC* and *sinH*. The *avrA* gene encodes a multifunctional protein that influences inflammation, epithelial apoptosis, and proliferation by altering the ubiquitination and acetylation of target proteins in host cells (Lu et al., 2014). Furthermore, the *iroC* gene is part of the *iroBCDEN* locus, which plays a vital role in the iron acquisition pathway of certain bacteria, enhancing their virulence and ability to colonize the host by transporting essential glucosylated siderophores (Crouch et al., 2008). In this study, the *cdtB* gene was detected in 72.73% of the isolates. The high prevalence of this gene observed may be attributed to a combination of genetic, ecological, and epidemiological factors, including horizontal gene transfer, selection pressure, and the dissemination of virulent clones. In particular, certain environments such as hospitals, livestock farms, and the food chain may create conditions that favor the selection of more virulent strains. Strains harboring the *cdtB* virulence gene may gain an adaptive advantage, especially in host colonization and persistence.

Our findings highlighted that certain isolates carried seven different virulence genes and exhibited several AMR genes. The detection of such bacteria may potentially be a serious problem for the patient and their contacts, as there is a risk of propagation. Furthermore, these findings should inform farmers, breeders, and public health practitioners including health and sanitation officials, and community town hall workers, to take necessary measures to ensure good hygiene, livestock, and agricultural practices for the general public. Findings from this study will also provide local data to relevant authorities on NTS prevalence and AMR genes from a one health perspective, which is a collaborative, multisectoral, and transdisciplinary approach, recognizing the interconnection among animals, plants, people, and their shared environment, to fight against antimicrobial resistance.

## 5 Conclusion

This study highlighted the diversity of NTS serotypes isolated from animal products, human clinical and environmental sources, as well as their antibiotic resistance and virulence genes. Several serotypes were identified, of which *S.* Schwarzengrund ST96 and *S*. Give ST516 were the most prevalent. Genes conferring resistance to chloramphenicol, fosfomycin, macrolide, sulfamethoxazole, tetracycline, trimethoprim, fluoroquinolone and aminoglycosides were observed in three of the *Salmonella* isolates. Effective, integrated AMR surveillance strategies and innovative control measures are needed to improve the management and mitigation of MDR NTS infections Burkina Faso and across the African sub-region.

## Data Availability

The raw data supporting the conclusions of this article will be made available by the authors, without undue reservation. Genome sequences have been deposited into Genbank under Bioproject number PRJNA1454954.

## Footnotes

1 https://www.bioinformatics.babraham.ac.uk/projects/fastqc/.

2 https://github.com/OpenGene/fastp.

3 http://bioinf.spbau.ru/spades.

4 http://bioinf.spbau.ru/quast.

5 http://vicbioinformatics.com/.

6 https://github.com/tseemann/mlst.

7 https://www.ncbi.nlm.nih.gov/pathogens/antimicrobial-resistance/AMRFinder/.

8 https://github.com/phacnml/sistr_cmd.

9 https://github.com/denglab/SeqSero2.

10 http://www.iqtree.org.
