# Supplementary figures and images for: Preliminary genomic characterisation and antimicrobial resistance of non-typhoidal Salmonella isolates from Burkina Faso within a One Health framework
Source: Front Microbiol. 2026 Jul 2;17:1743556. doi: 10.3389/fmicb.2026.1743556 (PMC13372896; doi:10.3389/fmicb.2026.1743556)

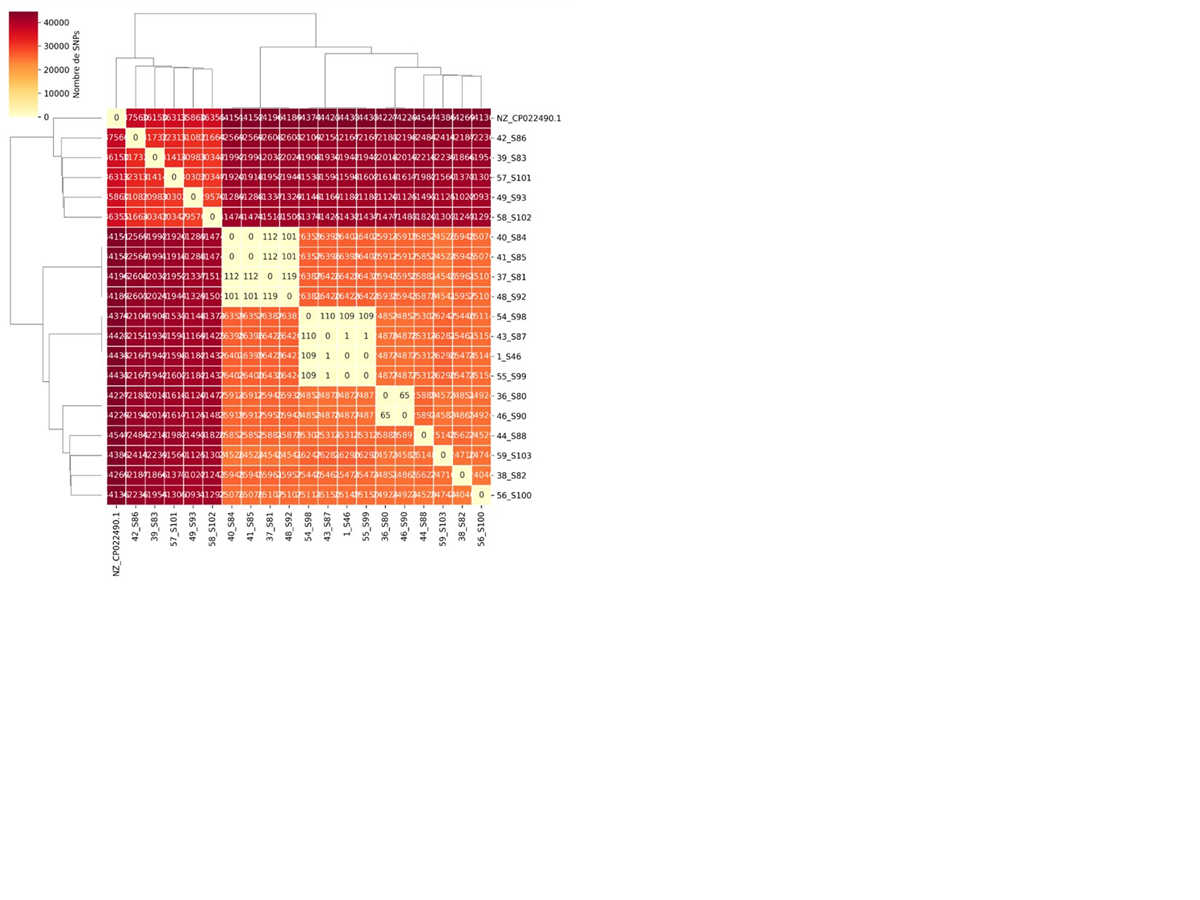

Supplement: Supplementary file 1 [file Image_1.tiff]
